# Colorectal Cancer: From Risk Factors to Oncogenesis

**DOI:** 10.3390/medicina59091646

**Published:** 2023-09-12

**Authors:** Vlad Alexandru Ionescu, Gina Gheorghe, Nicolae Bacalbasa, Alexandru Laurentiu Chiotoroiu, Camelia Diaconu

**Affiliations:** 1 Faculty of Medicine, University of Medicine and Pharmacy Carol Davila Bucharest, 050474 Bucharest, Romania; vladalexandru.ionescu92@gmail.com (V.A.I.); nicolae.bacalbasa@umfcd.ro (N.B.); 2 Internal Medicine Department, Clinical Emergency Hospital of Bucharest, 105402 Bucharest, Romania; 3 Department of Cellular and Mollecular Pathology, Stefan S. Nicolau Institute of Virology, Romanian Academy, 030304 Bucharest, Romania; 4 Gastroenterology Department, Clinical Emergency Hospital of Bucharest, 105402 Bucharest, Romania; 5 Department of Visceral Surgery, Center of Excellence in Translational Medicine, Fundeni Clinical Institute, 022328 Bucharest, Romania; 6 Department of Surgery, Clinical Emergency Hospital of Bucharest, 105402 Bucharest, Romania; chiotoroiu@yahoo.com; 7 Academy of Romanian Scientists, 050085 Bucharest, Romania

**Keywords:** colorectal cancer, risk factors, oncogenesis, lifestyle, gut microbiota, prognosis

## Abstract

Colorectal cancer is the second leading cause of cancer-related mortality worldwide. Numerous pathophysiological mechanisms, such as abnormal cell proliferation, cell differentiation, resistance to apoptosis, invasion of structures adjacent to colorectal tumor cells, and distant metastasis, are involved in colorectal carcinogenesis. These processes are initiated by the complex interaction of a number of genetic and environmental factors, including sedentary lifestyle, obesity, alcohol consumption, smoking, or gut microbiota. Despite the significant progress achieved in the diagnostic and therapeutic management of patients with colorectal cancer, there has been recently a noteworthy increase in the incidence of colorectal cancer in individuals below the age of 50 years. Early-onset colorectal cancer has a different frequency of oncogenic mutations, a higher prevalence of mucinous histology, a distinct deoxyribonucleic acid (DNA) methylation profile, a more distal location, and lower survival rates. A significant improvement in the prognosis of these patients can be achieved through the detection and removal of modifiable risk factors, along with the implementation of personalized screening strategies for individuals at high risk for this malignancy. Furthermore, gaining comprehension of the pathophysiological mechanisms by which these risk factors contribute to the process of oncogenesis may facilitate the discovery of novel therapeutic targets.

## 1. Introduction

Because of its high incidence and mortality rates, colorectal cancer poses a public health concern. In 2020, according to GLOBOCAN, colorectal cancer ranked third in the number of new cancer cases reported worldwide, with 1,931,590 cases (10%), and second in the number of fatalities associated with malignancies, with 935,173 deaths (9.4%) [1]. The geographic variation in incidence and mortality rates is closely related to the distribution of risk factors for this cancer [1]. Thus, Asia had the highest incidence of colorectal cancer (52.3%), followed by Europe (26.9%), North America (9.3%), Latin America and the Caribbean (7%), and Africa (3.4%). A total of 54.2% of colorectal cancer fatalities occurred in Asia, 26.2% in Europe, 7.4% in Latin America and the Caribbean, 6.8% in North America, and 4.8% in Africa [1]. The 5-year survival rate of patients with colorectal cancer is approximately 60% [2]. The International Agency for Research on Cancer (IARC) estimates that the number of new cases of colorectal cancer will increase by 63 percent, to 3.2 million per year by 2040, while the mortality rate will increase by 73 percent, to 1.6 million per year [2]. In addition, this increase in incidence rates has been found to be more pronounced among young adults and in nations undergoing economic transition [2]. With 7607 cases in 2020, colorectal cancer is the third most frequently reported malignancy among males in Romania, after lung cancer and prostate cancer [3]. With 5331 cases reported in 2020, this neoplasia ranks second in the list of new cancer cases in women, after breast cancer [3]. Colorectal cancer is the second leading cause of cancer-related mortality in Romania, accounting for 4302 fatalities in 2020 [3].

## 2. Oncogenesis in Colorectal Cancer

Colorectal carcinogenesis entails a series of pathophysiological mechanisms, such as abnormal cell proliferation, cell differentiation, resistance to apoptosis, invasion of adjacent structures by colorectal tumor cells, and distant metastasis [4,5]. A variety of genes and the interaction of multiple signaling pathways have been implicated in colorectal oncogenesis, but this complex mechanism remains incompletely understood [6,7]. A significant proportion of colorectal cancer cases are sporadic and develop slowly over several years within an adenoma–carcinoma sequence, despite the importance of the hereditary component [8]. Approximately 10% of adenomatous polyps evolve to adenocarcinoma, with the risk directly proportional to polyp size [9]. The development and progression of colorectal cancer are caused by the accumulation of mutations in the Wnt, epidermal growth factor receptor (EGFR), P53, and transforming growth factor beta (TGF-beta) signaling pathways [8,10,11]. The involvement of the Wnt signaling pathway has been demonstrated in oncogenesis, as well as in certain physiological processes, including cell differentiation, ocular angiogenesis, bone formation, or development of the central nervous system [10]. The abnormal activity of the Wnt/β-catenin signaling pathway promotes the proliferation and differentiation of cancer cells, playing a key role in colorectal oncogenesis and response to treatment [12]. The promotion of carcinogenesis by EGFR involves the dysregulation of the cell cycle, the stimulation of cell proliferation and angiogenesis, and the suppression of apoptosis via phosphorylation and inactivation of the proapoptotic Bcl-2-associated death promoter (BAD) protein [11].

Mutations in the adenomatous polyposis coli (APC) gene occur early in approximately 70% of patients with colorectal adenomas [8,13]. However, their progression to carcinomas is contingent on acquiring activating mutations of the KRAS oncogene and subsequent inactivating mutations of the tumor suppressor genes TP53 and SMAD4 [8]. The presence of APC mutation has been found to be correlated with a decreased tumor mutation burden (TMB), increased tumor purity (TP), reduced expression of immune checkpoint molecules (PD-a, PD-L1, PD-L2), decreased microsatellite instability, and an up-regulated mismatch repair pathway [13]. All of these factors are associated with an inadequate response to immunotherapy [13].

Oncogenesis is initiated in a small proportion of sporadic colorectal malignancies by activation of BRAF gene mutations, inactivation of genes involved in deoxyribonucleic acid (DNA) repair, and microsatellite instability (MSI) [8]. Several genetic syndromes, most of which are autosomal dominant, are associated with an elevated incidence of colorectal cancer. Among these, there are familial adenomatous polyposis (FAP), hereditary non-polyposis colorectal cancer (Lynch syndrome), polyposis associated with MUTYH gene mutations, Gardner syndrome, Turcot syndrome, juvenile polyposis syndrome, Peutz–Jeghers syndrome, and Cowden syndrome [9,14]. FAP and Lynch syndrome are the most prevalent familial colorectal cancer syndromes, accounting for approximately 5% of colorectal tumors [14]. Apart from syndromes with defined genetic predisposition, a family history of colorectal cancer is also associated with an increased risk of developing this cancer [14,15]. Thus, up to 30% of patients with colorectal cancer have a family history, and the risk of developing this malignancy in an individual with a family history of colorectal cancer in a first-degree relative is 2 to 4 times higher than in the general population [8,14]. In addition, individuals with two affected first-degree relatives or those with a first-degree relative diagnosed with colorectal cancer before the age of 50 years are at an increased risk [14].

Recent exhaustive transcriptomic analyses have established a consensus molecular classification (CMS) of colorectal cancer that goes beyond these genetic events (Table 1) [16]. 

In the past fifteen years, research has suggested that metabolic reprogramming, an active process governed by oncogenes and tumor suppressor genes, plays an essential role in the survival and multiplication of tumor cells [8,17]. Cancer cells utilize the majority of metabolic pathways involving glucose, glutamine, amino acids, serine/glycine, and lipids to sustain their high rates of cell division [8,18]. Beyond cell proliferation, the relationship between cell metabolism and cancer cell phenotype is becoming increasingly evident [8]. This can be explained by epigenetic alterations in the tumor microenvironment, as well as by the interaction of tumor cells with the surrounding microenvironment, including interactions with other cancer cells, stromal cells, and immune cells [8]. In addition, these metabolic interactions appear to play a crucial role in regulating tumor progression and determining chemotherapy response [8].

In recent years, early-onset colorectal cancer has been the subject of significant research interest. The implementation of screening programs has enhanced the early diagnosis of colorectal cancer in patients over the age of 50 years and reduced the global burden associated with this condition [19,20]. However, the proportion of patients diagnosed with colorectal cancer before the age of 50 years has consistently increased over the past decade [21,22]. Furthermore, epidemiological studies predict a 27.7% increase in early-onset colon cancer diagnoses and a 46% increase in rectal cancer diagnoses by 2030 [22]. From a molecular standpoint, colorectal cancer diagnosed before the age of 50 years has a different frequency of oncogenic mutations, a higher prevalence of mucinous (poorly differentiated) histology, a unique profile of DNA methylation, a more distal location, and lower survival rates [16]. Thus, a substantial decrease in the prevalence of mutations in the APC and Wnt signaling pathways was reported among patients with early cancer detection [16]. In contrast, mutations in the beta-catenin and CTNNB1 genes seem to be more frequent in these patients [16].

## 3. Risk Factors for Colorectal Cancers

**Figure 1:**
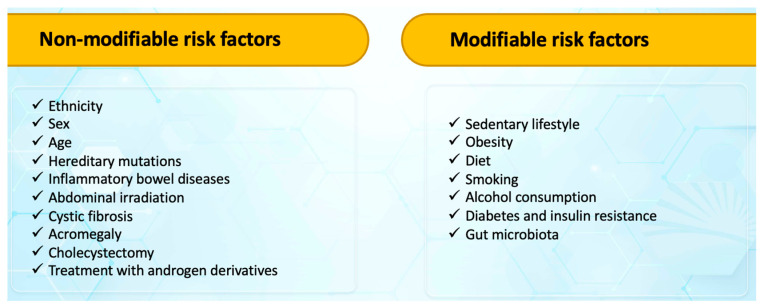
Risk factors for colorectal cancer.

There are two broad categories of colorectal cancer risk factors: non-modifiable and modifiable risk factors (Figure 1) [9,23,24,25]. 

Colorectal cancer is slightly more common in the black race than in the white race. This distinction appears to have more to do with access to health care or screening programs, diet, income, and education than with genetics [9]. In a recent study conducted by Meester et al., the authors observed a rise in the occurrence of early onset colorectal cancer across various racial groups. Notably, the study emphasized a more pronounced increase in the incidence of this malignant condition among individuals of non-Hispanic white ethnicity [26]. 

Men are approximately 1.5 times more likely than women to develop colorectal cancer, regardless of age or ethnicity [9]. In contrast, women are more likely to develop malignant diseases of the right hemi colon, with a more aggressive progression [9]. According to a recent meta-analysis comprising 3304 studies, it was observed an increase in the occurrence of early-onset colorectal cancer among males (relative risk 1.59, 95% confidence interval 1.23–2.07) [27].

In the United States, adults over the age of 65 years are three times more likely to develop colorectal cancer than those aged 50 to 64 years, and thirty times more likely than those aged 25 to 49 years [9]. However, recent epidemiological studies have documented a decline in the incidence of this disease among individuals older than 50 years and an increase among those younger than 50 years [9]. This may indicate a more sedentary lifestyle and has resulted in the implementation of screening strategies beginning at age 45 [9].

Compared to the general population, patients with inflammatory bowel diseases are twice as likely to develop colorectal cancer [9]. Chronic intestinal inflammation, which is a characteristic of these conditions, results in an aberrant release of cytokines and metabolic products, as well as an increase in local blood flow, all of which promote carcinogenesis [9].

There is a well-documented association between ulcerative colitis (UC) and an increased risk of colorectal cancer, with disease activity, severity, and duration serving as the primary determinants [28,29,30,31]. Consequently, pancolitis is associated with a 5- to 15-fold increased risk of colorectal cancer, whereas disease limited to the left colon is associated with a 3-fold increased risk compared to the general population [28]. In contrast, it does not appear that proctitis or proctosigmoiditis significantly increase the risk of colorectal cancer [28]. The incidence of colorectal cancer is reasonably estimated to be 0.5% per year for patients with a disease duration between 10 and 20 years, and 1% per year afterwards [28]. Most studies indicate that the presence of primary sclerosing cholangitis increases the risk of colorectal cancer. Large pseudopolyps or colonic strictures are additional elements that should raise the suspicion of a malignant progression of UC [28,30].

Less conclusive evidence exists regarding the association between Crohn’s disease and colorectal cancer risk [32,33,34,35]. In a recent study that followed 47,035 patients with Crohn’s disease for a median of more than 10 years, Olen et al. reported that 499 of them developed colorectal cancer [32]. In addition, 296 patients with colorectal cancer passed away [32]. These authors found no differences in the stage of colorectal cancer at the time of diagnosis between Crohn’s disease patients and a control group [32]. This study concluded that patients with Crohn’s disease have an increased incidence of colorectal cancer and death through cancer [32]. Patients diagnosed with Crohn’s disease before the age of 40 years, those with primary sclerosing cholangitis, and those with localized inflammatory lesions in the colon were also found to have a modestly increased risk [32].

A study that tracked individuals diagnosed with early onset colorectal cancer found that patients with a history of inflammatory bowel diseases exhibited higher occurrences of metastatic disease, a reduced degree of histological differentiation, and increased presence of lymphovascular and perineural invasion, in comparison to those with sporadic cancer [36]. Additionally, these patients experienced lower rates of survival, with statistical significance at a *p*-value of less than 0.0001 [36]. The prognosis did not differ between ulcerative colitis and Crohn’s disease, but it was worse for individuals with undifferentiated inflammatory bowel disease (*p* = 0.049) [36]. These authors hypothesized that the chronic inflammation associated with inflammatory bowel diseases could result in a specific genetic profile, which could explain the poor prognosis of this malignant condition [36]. They also noted a higher prevalence of mucinous and signet ring histology (negative prognostic factors) in the inflammatory bowel disease population, as well as higher rates of positive surgical margins [36]. 

Patients with a history of abdominal irradiation are at an increased risk of developing gastrointestinal neoplasms, most commonly colorectal cancer [37,38,39]. There is no consensus regarding the optimal time to initiate a colorectal cancer screening strategy or the optimal interval between assessments in pediatric cancer survivors. The guidelines of the Pediatric Oncology Group recommend colonoscopy every five years for survivors of radiation-treated childhood cancer, with the screening program beginning at the age of 30 years, or five years after the cessation of radiation therapy [40]. A history of radiotherapy for prostate cancer is associated with an increased risk of colorectal cancer comparable to that observed in patients with a family history of colonic adenomas [39,41]. It is unclear whether, in these cases, the adenoma–carcinoma sequence is followed or whether intensifying the screening strategy for colorectal cancer would increase the early detection rate for this condition [39]. Unlike adult survivors of childhood cancer, increased surveillance is not currently recommended in this group [39].

Compared to the general population, cystic fibrosis is associated with a 10-fold increased risk of colorectal cancer, and cholecystectomy is associated with an increased risk of right colon cancer [42,43,44]. Multiple hypotheses have been proposed regarding the pathogenesis of cancer in patients with cystic fibrosis [42,43,44]. One of these is the increased turnover of gastrointestinal epithelial cells in cystic fibrosis patients since early infancy [42,43]. CFTR gene mutations cause an increase in the viscosity of luminal secretions, impaired mucociliary clearance, and a predominantly neutrophilic inflammatory infiltrate [42,43]. Chronic inflammation can directly harm epithelial cells or lead to bacterial dysbiosis, resulting in the initiation of oncogenesis [42,45]. Than et al. observed a correlation between CFTR underexpression and the increased incidence of colon cancer, specifically the dysregulation of genes associated with immune responses, intestinal stem cells, and other regulators of signaling pathways that modulate cell growth [46]. Frequent radiation exposure (X-rays and repeated CT scans) may also contribute to the increased risk of cancer in cystic fibrosis patients [42]. Lagergren et al. claimed that bile has a damaging effect on the intestinal mucosa [47]. These authors hypothesized that bacterial degradation of bile salts into secondary bile acids could be pathogenic for the intestine, resulting in mucosal injury, cell proliferation, and eventually invasive cancer [47]. Another recently published population study found a larger incidence of CFTR mutations in colorectal cancer patients than in the general population [45]. In addition, the average age at which colorectal cancer was diagnosed was lower in populations with CFTR mutations than in populations without these mutations (52 years versus 73 years, *p* 0.01) [45].

Using the dietary inflammatory index, foods can be divided into two categories:

- pro-inflammatory diets are associated with an increased risk of colorectal cancer (carbohydrates, proteins, trans fats, cholesterol, saturated fatty acids, iron, etc.).
- foods with anti-inflammatory potential—associated with a lower risk of colorectal cancer (fibers, monounsaturated fatty acids, polyunsaturated fatty acids, omega 3, omega 6, niacin, thiamine, vitamin B6, vitamin B12, selenium, zinc, magnesium, fat-soluble vitamins (A, D, E, K), beta-carotene, folic acid, caffeine, tea, etc.) [48,49,50].

According to epidemiological data, there is a correlation between obesity and colorectal cancer [51,52,53,54]. Adipocytes within the microenvironment of a tumor are a source of energy that supports the proliferation of cancer cells [51,54]. Adipose tissue contributes to the appearance and development of colorectal cancer by increasing the secretion of adipokines, proinflammatory cytokines (interleukin-6, tumor necrosis factor alpha, plasminogen activator inhibitor 1—PAI-, etc.), insulin or insulin-like growth factor (IGF), respectively, the decrease in adiponectin secretion [51,52,54]. By activating signaling pathways that promote tumor cell proliferation and metastasis, inflammation plays a significant role in oncogenesis [22]. In addition, it has been shown that the serum level of estrogen is elevated in obese patients, but its role in the etiopathogenesis of colorectal cancer is controversial [54,55]. Another consequence of obesity is intestinal dysbiosis [55]. Thus, an increase in pathogenic microorganisms and metabolites (lipopolysaccharides) and a decrease in beneficial microbiota and metabolites (short-chain fatty acids) were observed [55]. Obese patients were found to have modestly increased levels of total bile acids, as well as significantly increased plasma and intrahepatic levels of conjugated bile acids and deoxycholic acid [55]. Numerous investigations have documented the role of bile acids in the induction of colorectal oncogenesis through the destruction of epithelial cells and stimulation of inflammatory processes [56,57]. All of these mechanisms may contribute to the progression and development of colorectal cancer [51].

Alcohol consumption is another risk factor for this cancer [58,59,60]. In a meta-analysis of nine cohort studies, Cai et al. found that the quantity of alcohol consumed was directly proportional to the increased risk of death from colorectal cancer [61]. Additionally, this correlation has been demonstrated to be stronger among Asians than among whites [58,60]. This phenomenon can be explained by genetic factors that regulate alcohol metabolism, as well as dietary factors, folate consumption, and body composition [58,59]. There is evidence that acetaldehyde, a metabolite of ethanol, plays a carcinogenic role by interfering with DNA synthesis and repair, modifying glutathione structure and function, and stimulating colonic mucosal proliferation [58,59].

The association between smoking and colorectal cancer risk is now well-established [62,63,64]. In a recent study of 188,052 subjects, Gram et al. reported a 39% increased incidence of left colon cancer in male smokers compared to nonsmokers [62]. In contrast, female smokers had a 20% higher risk of developing right colon cancer as opposed to nonsmokers [62]. Compared to male smokers, female smokers have a higher risk of developing rectal cancer, with the risk being directly proportional to the number of packs per year [62]. More than 7000 toxic chemicals, about 70 of which are known carcinogens, are inhaled by smokers. By inhaling them directly or hematogenous dissemination, nitrosamines, heterocyclic amines, polycyclic aromatic hydrocarbons, and benzene can reach the colorectal mucosa and exert a local pro-oncogenic effect [65]. The evidence regarding the relationship between smoking and the presence of colorectal adenomas suggests that this detrimental behavior plays a role in early carcinogenesis [65]. Another study evaluating molecular changes in smokers found elevated levels of microsatellite instability, BRAF gene mutations, and the CpG island methylator phenotype (CIMP) [66]. Furthermore, it has been observed that smoking is associated with an additional increase in the risk of acquiring colorectal adenomas in patients diagnosed with Lynch syndrome [67]. Winkels et al. reported a hazard ratio of 6.13 for the development of colorectal adenomas in smoking patients with Lynch syndrome, as compared to those who do not engage in smoking behavior [67]. The aforementioned study observed a tendency towards a heightened incidence of colorectal adenomas in individuals with Lynch syndrome who engage in alcohol consumption [67]. 

Diabetes is a risk factor for colorectal cancer regardless of body mass index, physical activity, or smoking status [68,69,70]. The pathophysiological mechanism underlying this correlation appears to be the increase in serum levels of insulin and IGF-1, with the mitogenic action of these biomarkers promoting the proliferation of colorectal epithelial cells [71]. A meta-analysis published in 2022 highlighted the increased risk of colorectal cancer among diabetic patients [71]. Compared to the left hemicolon, these patients were 13% more likely to develop colon cancer in the right hemicolon [71]. These findings could have significant implications for colorectal cancer screening programs in diabetic patients [31]. Therefore, in this subpopulation, it is imperative to evaluate the colon’s proximal segments [71]. Moreover, diabetes appears to affect the prognosis of colorectal cancer patients [72]. In a study of 2278 patients with nonmetastatic colorectal cancer, Dehal et al. found a direct proportional relationship between diabetes and the risk of mortality from all causes, respectively, the risk of death associated with this cancer [73]. Another meta-analysis that included 10 million individuals raised the hypothesis that the increased prevalence of diabetes among young adults could contribute to the increased incidence of early-onset colorectal cancer [74]. 

Through direct interaction with enterocytes and participation in processes such as cellular metabolism and immune response, the intestinal microbiome is also involved in colorectal oncogenesis [75,76]. Sequencing studies have revealed alterations in the composition of the intestinal microbiota, as well as a reduction in its diversity, in colorectal cancer patients [75,77]. Thus, a predominance of species including *Bacteroides fragilis*, *Alistipes finegoldi*, *Fusobacterium nucleatum*, *Parvimonas micra*, *Porphyromonas asaccharolytica*, *Prevotela intermedia*, and *Thermanaerovibrio acidaminovora* was detected [75,78]. Moreover, several studies have provided evidence for the presence of a distinction in the microbial composition between the tumor site and the remaining sections of the intestinal microbiome [79,80,81,82]. At the level of tumor tissue, an abundance of bacteria belonging to the genera Dialster, Fusobacterium, Campylobacter, and Gemella was observed, while the presence of Blautia and Allistipes was found to be comparatively lower. [79]. Therefore, the analysis of the tumor microbiome revealed an elevated presence of pathogenic bacteria, which is often linked to a higher susceptibility to colorectal cancer. Conversely, the microbiome composition in normal intestinal tissue primarily consisted of saprophytic bacteria that produce short-chain fatty acids [83,84,85]. Furthermore, Debelius et al. reported a disparity in the composition of the tumor microbiome compared to the microbiome of healthy tissue, specifically in colorectal cancer patients with a shorter lifespan [79]. This discrepancy was not observed in patients with long-term survival, indicating the potential prognostic significance of the gut microbiome in individuals diagnosed with colorectal cancer [79]. These results have been confirmed by another study, which documented a significant association between the prevalence of the genera Fusobacterium and Bacteroides with decreased overall survival, and conversely, an association between Faecalibacterium and shorter-term survival [86]. 

Patients with early-onset colorectal cancer were found to show an elevated degree of microbial diversity [87]. In patients with young-onset colorectal cancer, *Flavonifractor plautii* was found to be the prevalent microbial population, as opposed to Streptococcus in patients with old-onset colorectal cancer [87]. In addition, DNA binding and RNA-dependent DNA biosynthetic process pathways were overrepresented in patients with early-onset colorectal cancer, indicating increased proliferation and invasion rates [87]. Tumor cells adapt their metabolic processes to fulfill their requirements for macromolecules, facilitate their rapid proliferation, and effectively counteract oxidative stress [88]. Hence, it was observed that the pentose phosphate pathway was stimulated, resulting in an augmentation of nucleotide generation and DNA synthesis [88]. These findings resulted in a decrease in the intracellular concentration of reactive oxygen species and an enhancement of antioxidant defense mechanisms [88]. In summary, the distinct microbiota present in individuals with early-onset colorectal cancer has been found to be linked to metabolic alterations that contribute to the advancement of the malignant condition and unfavorable prognosis [88].

## 4. Future Research Directions

The comprehension of the physiopathological mechanisms by which specific risk factors contribute to the development of colorectal cancer can facilitate the discovery of novel biomarkers that play a role in early detection of this condition. The sensitivity of fecal tests utilized in screening programs for colorectal cancer is limited (50–75% for high sensitivity guaiac fecal occult blood test and 74% for fecal immunochemical test) [89]. The multi-target stool DNA test, which enables the detection of 11 molecular biomarkers, is associated with significant expenses and a considerable number of false positive outcomes (specificity of 85% for colorectal cancer) [89]. However, screening programs employing colonoscopy, which is considered “gold standard” diagnostic method for colorectal cancer, encounter challenges in terms of patient adherence. This is attributable mostly to the discomfort involved with the inquiry’s preparation, but it also arises from the investigation itself. In colorectal cancer screening programs, computed tomographic colonography is another diagnostic technique that can be utilized. However, this entails radiation exposure, indeterminate findings in 1.3–11.4 percent of cases, and the need to continue diagnostic management with a colonoscopy if results are suggestive of adenomatous polyps or colorectal cancer [89]. Despite the considerable amount of research conducted in these domains and the encouraging outcomes observed for novel screening approaches, such as liquid biopsy, stool-based microbiome testing, or urine-based screening tests, none of these methods have received approval for implementation in present-day clinical practice [89]. Under these circumstances, the discovery of certain non-invasive and economically viable biomarkers that play a role in the timely detection of colorectal cancer has the potential to enhance the prognosis of individuals affected by this disease.

Moreover, in light of the increasing prevalence of early-onset colorectal cancer, the development of individualized screening approaches grounded on specific risk factors has the potential to enhance the prognosis of these patients. According to the most recent guideline issued by the American College of Gastroenterology, it is suggested to start the implementation of a screening strategy for colorectal cancer among individuals in the general population at the age of 45 [90]. At now, there exist personalized screening strategies for individuals diagnosed with specific genetic disorders; nevertheless, it is important to note that this subset constitutes a relatively minor fraction of the overall population of colorectal cancer patients. The development of cost-effective and non-invasive biomarkers with high accuracy in diagnosis for early-stage colorectal adenomas or carcinomas is crucial. These biomarkers would facilitate the identification of patients who might benefit from colonoscopy, thereby enhancing the long-term morbidity and death outcomes associated with colorectal cancer. In patients with certain modifiable risk factors for this malignant disease, the implementation of the screening strategy at an earlier age could improve early detection rates of early-onset colorectal cancer and, secondarily, the prognosis, but also the costs involved in the medical care of these patients.

**Figure 2:**
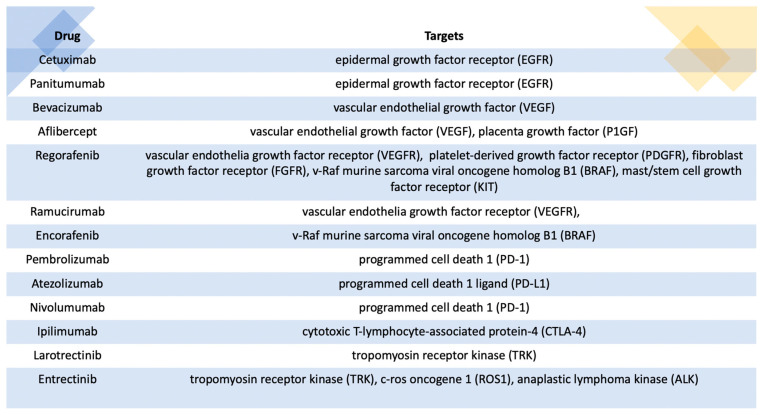
Targeted therapy for colorectal cancer.

Another avenue of research that stems from comprehending the physiopathological mechanisms involved in colorectal oncogenesis pertains to the discovery of novel therapeutic targets that facilitate the advancement of targeted therapeutics. Over the past few decades, there has been significant progress in the development of medications that specifically target signaling pathways associated with carcinogenesis as well as the tumor microbiome (Figure 2) [91,92]. However, a large number of patients experience disease progression, even under these targeted therapeutic regimens [91]. Given the existing statistical data indicating a 5-year survival rate of roughly 60% for individuals diagnosed with colorectal cancer, it becomes crucial to develop novel therapeutic agents that can enhance the prognosis of these patients [2].

A close collaboration between molecular biologists and clinical practitioners is required to further elucidate colorectal cancer mechanisms and enhance patient outcomes in light of these considerations. Furthermore, it is of greatest need to widely distribute this information to both the general population, with the aim of raising their awareness of the importance of adhering to screening programs and embracing a healthy lifestyle, as well as to primary care physicians, in order to guide patients towards appropriate screening programs.

## 5. Conclusions

The tumorigenesis of colorectal cancer can be caused by the activation of various proto-oncogenes, the inactivation of some tumor suppressor genes, chromosomal instability, microsatellite instability, as well as epigenetic alterations in the DNA. The genetic alterations mentioned above interact with various environmental factors, including a diet high in saturated fats and carbohydrates, a sedentary lifestyle, obesity, smoking, or alcohol consumption, to initiate the process of oncogenesis. The implementation of screening programs enhanced the early detection of colorectal cancer in patients over the age of 50 years and contributed to the reduction of morbidity and mortality rates associated with this condition. However, the proportion of patients diagnosed with colorectal cancer before the age of 50 years has consistently increased over the past decade. When considering colorectal cancer from a molecular perspective, it is observed that there are variations in the frequency of oncogenic mutations between cases diagnosed before and after the age of 50 years. Additionally, those diagnosed before this age exhibit a higher prevalence of mucinous (poorly differentiated) histology, a unique DNA methylation profile, a greater tendency for distal location, and lower survival rates. Despite the impressive advances in the diagnostic and therapeutic management, the poor prognosis of colorectal cancer patients highlights the need for a better understanding of the mechanisms that contribute to the disease’s initiation and progression. 

Increased knowledge regarding risk factors for colorectal cancer has the potential to enhance the vigilance of physicians and their patients towards early symptoms of this malignancy, such as weight loss, rectal bleeding, alterations in bowel habits, abdominal pain, and iron deficiency. In conclusion, it is imperative to utilize these identified risk factors in the development of straightforward predictive models for early-onset colorectal cancer. These models can then be employed to implement targeted screening strategies for high-risk populations, thereby effectively decreasing the occurrence of early-onset colorectal cancer without necessitating a universal adjustment in the recommended age for screening. 

## Figures and Tables

**Table 1:** The consensus molecular classification (CMS) of colorectal cancer [9].

Classification	Description
CMS 1	associated with activation of the JAK-STAT signaling pathway, micro-satellite instability, and hypermutated tumor DNA
CMS 2	associated with activation of the Wnt/MYC signaling pathway
CMS 3	associated with metabolic changes
CMS 4	associated with epithelial–mesenchymal transition and immunosuppression
Consensus molecular classification (CMS); Janus kinase/signal transduction and transcription activation (JAK-STAT); Wingless-related integration site/myelocytomatosis oncogene (Wnt/MYC).	

## Data Availability

No new data were created or analyzed in this study. Data sharing is not applicable to this article.
